# miRNA expression profiling of hereditary breast tumors from *BRCA1*- and *BRCA2*-germline mutation carriers in Brazil

**DOI:** 10.1186/s12885-020-6640-y

**Published:** 2020-02-22

**Authors:** Danielle Pessôa-Pereira, Adriane Feijó Evangelista, Rhafaela Lima Causin, René Aloisio da Costa Vieira, Lucas Faria Abrahão-Machado, Iara Viana Vidigal Santana, Vinicius Duval da Silva, Karen Cristina Borba de Souza, Renato José de Oliveira-Silva, Gabriela Carvalho Fernandes, Rui Manuel Reis, Edenir Inêz Palmero, Márcia Maria Chiquitelli Marques

**Affiliations:** 1 Molecular Oncology Research Center, Barretos Cancer Hospital, Barretos, SP Brazil; 2 Department of Breast and Reconstructive Surgery, Barretos Cancer Hospital, Barretos, SP Brazil; 3 Department of Pathology, Barretos Cancer Hospital, Barretos, SP Brazil; 4 Life and Health Sciences Research Institute (ICVS), Medical School, University of Minho, Braga, Portugal; 5 ICVS/3B’s-PT Government Associate Laboratory, Braga/Guimarães, Portugal; 6 Center of Molecular Diagnosis, Barretos Cancer Hospital, Barretos, SP Brazil; 7 Department of Oncogenetics, Barretos Cancer Hospital, Barretos, SP Brazil; 8 Barretos School of Health Sciences, Dr. Paulo Prata – FACISB, Barretos, SP Brazil; 9 Tumor Biobank, Barretos Cancer Hospital, Barretos, SP Brazil

**Keywords:** microRNA, Biomarker, NanoString, Hereditary breast tumors

## Abstract

**Background:**

MicroRNAs (miRNAs) are small non-coding RNAs involved in post-transcriptional gene expression regulation and have been described as key regulators of carcinogenesis. Aberrant miRNA expression has been frequently reported in sporadic breast cancers, but few studies have focused on profiling hereditary breast cancers. In this study, we aimed to identify specific miRNA signatures in hereditary breast tumors and to compare with sporadic breast cancer and normal breast tissues.

**Methods:**

Global miRNA expression profiling using NanoString technology was performed on 43 hereditary breast tumors (15 BRCA1, 14 BRCA2, and 14 BRCAX), 23 sporadic breast tumors and 8 normal breast tissues. These normal breast tissues derived from *BRCA1*- and *BRCA2*- mutation carriers (*n* = 5) and non-mutation carriers (*n* = 3). Subsequently, we performed receiver operating characteristic (ROC) curve analyses to evaluate the diagnostic performance of differentially expressed miRNAs. Putative target genes of each miRNAs considered as potential biomarkers were identified using miRDIP platform and used for pathway enrichment analysis.

**Results:**

miRNA expression analyses identified several profiles that were specific to hereditary breast cancers. A total of 25 miRNAs were found to be differentially expressed (fold change: > 2.0 and *p* < 0.05) and considered as potential biomarkers (area under the curve > 0.75) in hereditary breast tumors compared to normal breast tissues, with an expressive upregulation among BRCAX cases. Furthermore, bioinformatic analysis revealed that these miRNAs shared target genes involved in ErbB, FoxO, and PI3K-Akt signaling pathways.

**Conclusions:**

Our results showed that miRNA expression profiling can differentiate hereditary from sporadic breast tumors and normal breast tissues. These miRNAs were remarkably deregulated in BRCAX hereditary breast cancers. Therefore, miRNA signatures can be used as potential novel diagnostic biomarkers for the prediction of *BRCA1/2*- germline mutations and may be useful for future clinical management.

## Background

Breast cancer is the most commonly diagnosed cancer among women worldwide after non-melanoma skin cancer and the leading cause of cancer-related deaths in developing countries [1, 2]. Although most breast tumors arise due to acquired mutations caused mainly by lifestyle and environmental factors, approximately 5 to 10% are attributable to inherited pathogenic variants in cancer-predisposing genes [3, 4]. Indeed, breast tumors have been reported within the tumor spectrum of many hereditary cancer syndromes [5]. However, the most common entity linked to inherited breast cancer is the hereditary breast and ovarian cancer (HBOC) predisposition syndrome, which is a highly penetrant, autosomal dominant condition primarily caused by germline pathogenic variants in breast cancer type 1 and 2 susceptibility genes (*BRCA1* and *BRCA2*) [3–6]. Although 50% of suggested HBOC cases are of unknown genetic origin (also termed ‘BRCAX’), pathogenic variants in *BRCA1/2* are more frequent (9–29%) than pathogenic variants in other high-penetrance genes (4–11%) [7, 8].

*BRCA1* and *BRCA2* are well-known tumor suppressor genes (TSGs) involved in many cellular processes implicated in the maintenance of genome integrity. Therefore, pathogenic variants in *BRCA1* or *BRCA2* can disrupt important biological functions, allowing the accumulation of genetic alterations and consequently increasing cancer susceptibility [9, 10]. Indeed, female individuals who carry a *BRCA1/2*-germline variant have a lifetime risk of developing breast cancer of up to 87% [11]. This may present a high histologic grade and, in particular for *BRCA1*-mutation carriers, a high mitotic index and triple-negative phenotype [12]. The identification of *BRCA1/2*-pathogenic variants is imperative and could directly impact on prevention, early cancer diagnosis, and clinical management of patients.

Genetic counseling and testing for *BRCA1/2*-germline mutations are currently available; however, screening of these mutations is still expensive and time-consuming because both genes do not present mutational hotspot regions; thus, such genetic alterations can occur throughout all the coding sequences [13]. Accordingly, many prediction models have been developed and are widely used to estimate the pre-test likelihood of identifying individuals and families at high risk for carrying these mutations [14–18]. Some studies that have evaluated the performance of the breast cancer genetic risk models reported low specificity rates for predicting *BRCA1/2*-germline mutations [19–22]. Therefore, there is a need to define additional parameters that could complement the current criteria adopted by the available prediction models to provide an accurate and effective selection of patients that should proceed to *BRCA1/2* genetic testing.

MicroRNA (miRNA) are small non-coding RNAs that could promote tumor development and/or progression by disturbing oncogenes and tumor suppression expression patterns [23–25]. Several studies have found distinctive miRNA expression profiles in a wide range of human tumors, suggesting that miRNA profiling could be used for diagnostic purposes [26–29]. An advantage of miRNAs is that they are more resistant to degradation caused by the formalin-fixed paraffin-embedded (FFPE) tissue processing [30]. However, little is known about miRNA expression in hereditary breast cancers (HBC) [31–33]. Moreover, it remains unclear whether miRNA profiling could be useful to distinguish *BRCA1/2*-mutation carriers from non-carriers.

In order to identify miRNA signatures that could serve as potential biomarkers to discriminate HBC, we evaluate the expression profiles of miRNAs in HBC tumors, sporadic breast cancer (SBC), and normal breast tissues (NBT) from carriers and non-carriers of *BRCA1* or *BRCA2* pathogenic germline mutations using NanoString technology. We demonstrate that miRNA expression profiles can discriminate HBC from SBC and BRCAX breast cancer. Therefore, these miRNAs could be useful as potential diagnostic biomarkers to improve the performance of the *BRCA1/2*-mutation prediction models and impact on the clinical management of breast cancer patients who may benefit from platinum-based chemotherapy and PARP inhibitors, such as olaparib [34].

## Methods

### Study population and clinicopathological features

A retrospective cohort study was performed in a total of 74 unrelated female patients admitted at Barretos Cancer Hospital between 2003 and 2017, including 66 patients with primary invasive breast cancer and 8 patients attended for reasons other than personal history of malignancy. Our cohort comprised 29 HBC patients harboring a confirmed *BRCA1* (*n* = 15) or *BRCA2* (*n* = 14) pathogenic germline mutation (a subset of cases derived from a larger population [35]); 14 HBC patients who were referred for *BRCA1*, *BRCA2, TP53* and *PTEN* genetic testing for meeting clinical criteria for HBOC, but no pathogenic variants were found – therefore considered as BRCAX; 23 SBC patients with no family history of breast and/or ovarian cancer; five healthy individuals harboring a *BRCA1* (*n* = 3) or *BRCA2* (*n* = 2) pathogenic germline mutation who had undergone prophylactic mastectomies; and three healthy patients with no family history of breast and/or ovarian cancer.

All patients belonging to high-risk HBOC families were referred by the Department of Oncogenetics of Barretos Cancer Hospital for genetic testing after fulfilling the clinical criteria defined by the National Comprehensive Cancer Network for a personal and/or family history of HBOC. Healthy *BRCA1/2*-mutation carriers were referred after a pathogenic germline mutation was identified in their families and were invited to undergo a mutation-specific predictive genetic test. All information regarding genetic counseling, genetic testing, and the management of the families at risk for hereditary cancer in our institution have been described in detail elsewhere [36].

### Pathological evaluation

All clinical and pathological data were collected from medical records. Histologic tumor grade was assessed by the modified Scarff-Bloom-Richardson grading system. Tumor staging was performed according to the seventh edition of the American Joint Committee on Cancer TNM system. Breast cancers were also classified into three intrinsic molecular subtypes (luminal, human epidermal growth factor receptor 2 (HER2)+, and triple-negative) based on the combined evaluation of estrogen receptor (ER), progesterone receptor (PR), and HER2 expression status according to the 13th St Gallen International Expert Consensus [37]. Evaluation of ER, PR, and HER2 status was done using FFPE sections as part of routine practice at the Pathology Department for breast cancer clinical assessment defined according to current guidelines [38–40].

### Sample collection and RNA isolation

FFPE breast tissue samples were obtained from the archives at the Department of Pathology of the Barretos Cancer Hospital. FFPE samples underwent total RNA isolation using the QIASymphony SP automated system based on magnetic-bead technology (QIAGEN, Hilden, Germany) according to the manufacturer’s protocol (RNA 130 FFPE). Quantification and RNA quality assessment were performed using a Nanodrop 2000 spectrophotometer (NanoDrop Products, Wilmington, DE, USA).

### NanoString nCounter miRNA assay

miRNA expression profiling was performed using the nCounter Human v3 miRNA Expression Assay Kit (NanoString Technologies, Seattle, WA, USA), according to the manufacturer’s protocol. Briefly, 100 ng of total RNA from each sample underwent sample preparation involving multiplexed annealing of specific tags onto the 3′ end of each mature miRNA, followed by a ligation reaction and an enzymatic purification to remove non-ligated tags. Next, miRNAs were hybridized with probe pairs which comprised biotin-labeled capture probes and fluorescent color-barcoded reporter probes for 21 h at 65 °C. For sample preparation and hybridization steps, a Veriti 96-Well Thermal Cycler (Applied Biosystems, Foster City, CA, USA) was used to ensure the temperature control required for the enzymatic reactions. Unhybridized probes were washed away using magnetic bead-based purification on the nCounter Prep Station (NanoString Technologies, Seattle, WA, USA). Purified target-probe complexes were subsequently eluted from the beads and immobilized on cartridges with streptavidin-covered surfaces. Finally, the cartridges were transferred into the nCounter Digital Analyzer (NanoString Technologies, Seattle, WA, USA) for data collection consisting of digital imaging and direct quantification of the individual fluorescent barcodes.

### NanoString data analysis

NanoString raw data were submitted to R version 3.6.1 (R Foundation, Vienna, Austria) and analyzed using the NanoStringNorm R package (version 1.1.21) [41]. Briefly, the following normalization steps were applied after probe-level background correction by code-count normalization using geometric mean parameter and sample content was normalized using the top 10 low Coefficient Vallue (CV) probes’ values. Normalized data were log2-based transformed and subsequently used as input for the differential expression analyses. The miRNAs differentially expressed were further filtered according to the presence in normal vs. sporadic group comparisons.

### Differential expression analysis

Statistical analysis of NanoString miRNA differential expression in R was performed using the Linear Models for Microarray Data (limma) package from Bioconductor. Limma has incorporated the most cutting-edge statistical analysis methods, providing functions for differential expression by empirical Bayes moderation of the standard errors. In the present study, it was used the moderated t-statistics for two-class comparisons and moderated F-statistics por multiple comparisons. It was considered as differentially expressed the miRNAs with FDR-corrected *p*-value less than 0.05, and a two-fold change difference in the expression levels between the groups evaluated (NBT vs SBC; NBT vs HBC (BRCA1, BRCA2 and BRCAX)).

### The receiver operating characteristic curves (ROC) curve analysis

ROC curve and the area under the curve (AUC-ROC) were used as a filter for the differentially expressed miRNAs. The ROCR R package in R environment was used to identify the true positive rate (sensitivity) as a function of the false positive rate (1-specificity) and the AUC values. The coord function of the pROC package was used to compute the best threshold values. In the present study, miRNAs presenting an AUC-ROC ≤0.75 were excluded and Table 2 shows all the associated values (sensitivity, specificity, AUC and cutoff values).

### Hierarchical clustering

Expression profiles of selected miRNAs (differentially expressed and filtered according to FDR-corrected *p*-values less than 0.05, fold-change greater than 2.0, absence in normal vs. sporadic group comparisons and AUC-ROC greater than 0.75) were grouped in order to evaluate their related expression patterns. It was used the hierarchical clustering as the clustering methodology with Euclidean distance to generate a hierarchical series of nested clusters represented graphically as dendrograms. The ComplexHeatmap package of Bioconductor was used for the generation of the related heatmaps. Red color indicates upregulation and green, downregulation.

### Target prediction and pathway enrichment analysis

We identified putative target genes of all differentially expressed miRNAs through the microRNA Data Integration Portal (mirDIP) (http://ophid.utoronto.ca/mirDIP/), a web-based computational database that integrates dozens of bioinformatic tools for miRNA target prediction. We restricted our search by considering predicted miRNA-target interactions under very high confidence (top 1%). From the obtained gene lists, we selected targets predicted by at least three of the following five prediction tools: DIANA, microrna.org, RNA22, RNAHybrid, and TargetScan. Considering the mechanism by which miRNAs downregulate their target genes and may impact on carcinogenesis and tumor progression, we only focused on genes previously described as TSGs or oncogenes in human cancers according to the Catalogue of Somatic Mutations in Cancer (COSMIC) (https://cancer.sanger.ac.uk/cosmic). The gene lists generated after applying all these selection criteria were used as input data for further analysis in Cytoscape (version 3.6.1), software for integration, visualization, and investigation of regulatory networks (https://cytoscape.org/). To evaluate whether co-expressed miRNAs could cooperatively affect breast cancer-related biological processes and pathways, we performed Gene Ontology (GO) enrichment analysis using the ReactomeFIViz app (version 7.0.1), a Cytoscape plugin that provides networks of functional regulatory interactions and curated biological pathways derived from Reactome and other databases [42]. For this purpose, only breast neoplasm-associated genes according to the Cancer Gene Index Annotations provided by the National Cancer Institute were selected for pathway enrichment and GO analyses through the Load Cancer Index function available in ReactomeFIViz. Only the process presenting at least three genes and an FDR-corrected *p*-value ≤0.05 was considered.

### Statistical analysis

Patient data were presented as frequencies and percentages for qualitative variables and the chi-square test or Fisher’s exact test were used to compare frequencies using the SPSS Statistics for Windows, version 20.0 (IBM, Armonk, NY, USA).

## Results

MiRNA expression profiling was performed on a total of 74 FFPE samples, which comprised 43 HBC (15 BRCA1; 14 BRCA2; 14 BRCAX), 23 SBC and 8 NBT from 3 *BRCA1*-mutation carriers, 2 *BRCA2*-mutation carriers and 3 non-carriers. Main demographics and clinicopathological characteristics of the population are showed in Table 1.

**Table 1:** Clinicopathological features of the patients included in the differential expression analyses

Characteristics	HBC			SBC	NBT	
	*BRCA1*	*BRCA2*	*BRCAX*	*n* = 23	*BRCA1/2*	WT
	*n* = 15	*n* = 14	*n* = 14		*n* = 5	*n* = 3
Clinical						
Age at diagnosis, y						
Mean (SD)	43.73 (8.30)	44.57 (11.18)	41.78 (12.14)	48.73 (10.45)	41.80 (5.89)	58.00 (9.16)
Range	29–59	26–67	25–66	30–77	35–51	50–68
Pathological, n (%)						
Grade (SBR)*						
.. .1	1 (6,7)	0	1 (7.1)	0		
.. .2	4 (26.7)	5 (35.7)	6 (42.9)	3 (13)		
... 3	10 (66.7)	9 (64.3)	7 (50)	20 (87)		
ER*						
Negative	12 (80)	4 (28.6)	5 (35.7)	17 (73.9)		
Positive	3 (20)	10 (71.4)	9 (64.3)	6 (26.1)		
PR*						
Negative	11 (73.3)	6 (42.9)	7 (50)	19 (82,6)		
Positive	4 (26.7)	8 (57.1)	7 (50)	4 (17.4)		
HER2 amplification*						
Negative	14 (93.3)	12 (85.7)	11 (78.6)	19 (82,6)		
Positive	1 (6.7)	2 (14.3)	3 (21.4)	4 (17.4)		
Molecular subtype*						
Luminal	4 (26.7)	11 (78.6)	9 (64.3)	6 (26.1)		
HER2+	0	0	2 (14.3)	1 (4.3)		
Triple-negative	11 (73.3)	3 (21.4)	4 (21.4)	16 (69.6)		
TNM*						
I	2 (13.3)	0	3 (21.4)	1 (4.3)		
II	9 (60)	4 (28.6)	9 (64.3)	11 (47.8)		
III	3 (20)	8 (57.1)	1 (7.1)	10 (43.5)		
IV	1 (6.7)	2 (14.3)	1 (7.1)	1 (4.3)		

(*) For breast tumors only.

*HBC*, hereditary breast cancer; *SBC*, sporadic breast cancer; *NBT*, normal breast tissue; *WT*, wild-type; y, years; *SD*, standard deviation; *SBR*, Scarff-Bloom-Richardson; *ER*, estrogen receptor; *PR*, progesterone receptor; *HER2*, human epidermal growth factor receptor 2.

### miRNA signatures of sporadic breast tumors

**Fig. 1:**
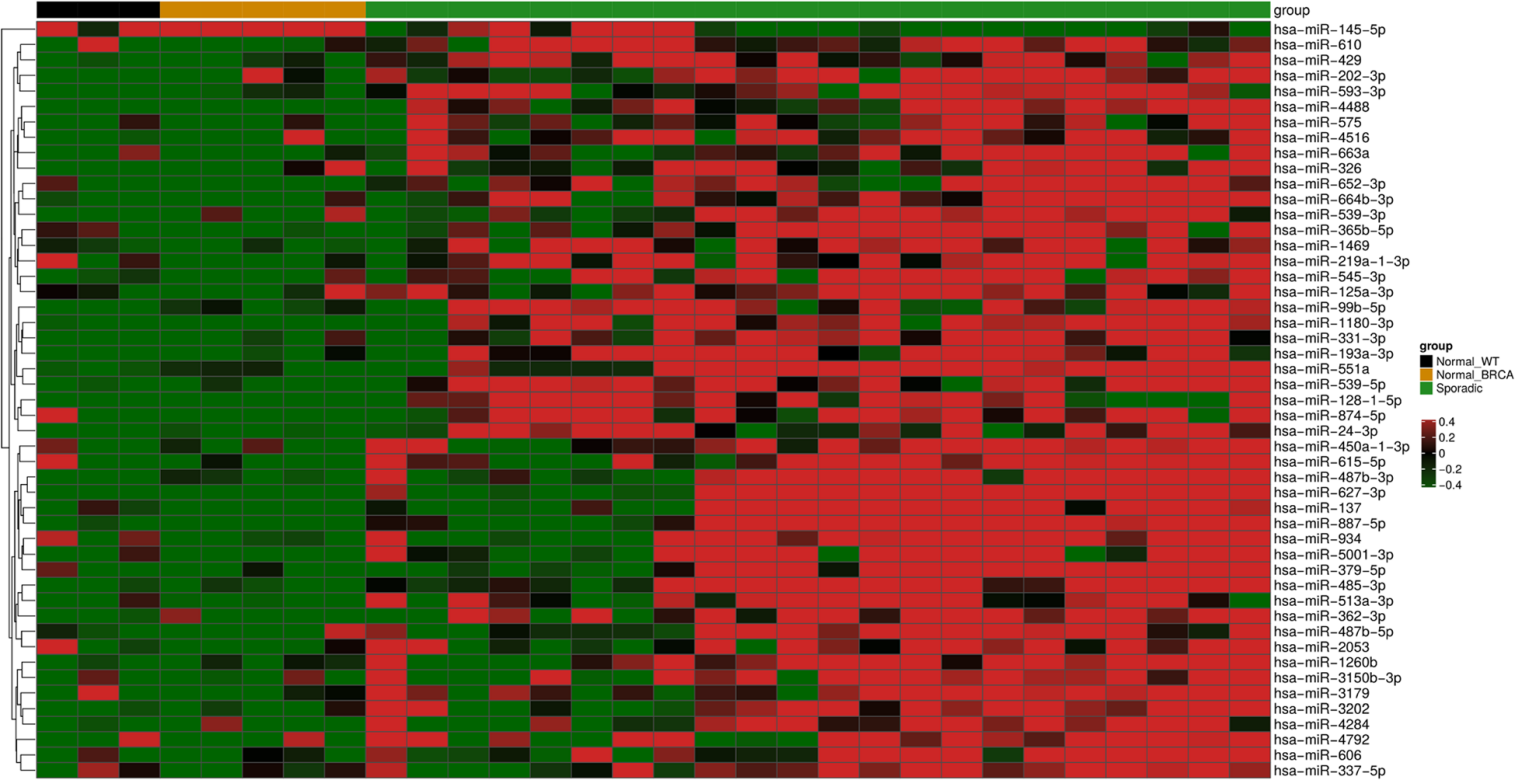
Heat map showing a supervised clustering of differentially expressed miRNAs between NBT and SBC. Each column indicates a sample and each row, a miRNA. Red color indicates upregulation and green, downregulation

In order to establish miRNA expression signatures specifically associated with HBC, we firstly investigated which miRNAs were significantly altered in SBC samples compared with NBT – which could allow us to identify whether any deregulated miRNAs are shared between the SBC and HBC groups later. We found a total of 49 miRNAs significantly upregulated (fold change values: ≥2.0; *p* value: < 0.05) in sporadic breast tumors as compared to NBT groups, yet no downregulated miRNAs were found (Fig. 1). This first analysis allowed us to identify whether any miRNAs are shared between the SBC and HBC groups.

### miRNA expression profiling of hereditary breast cancer and normal breast tissues

**Fig. 2:**
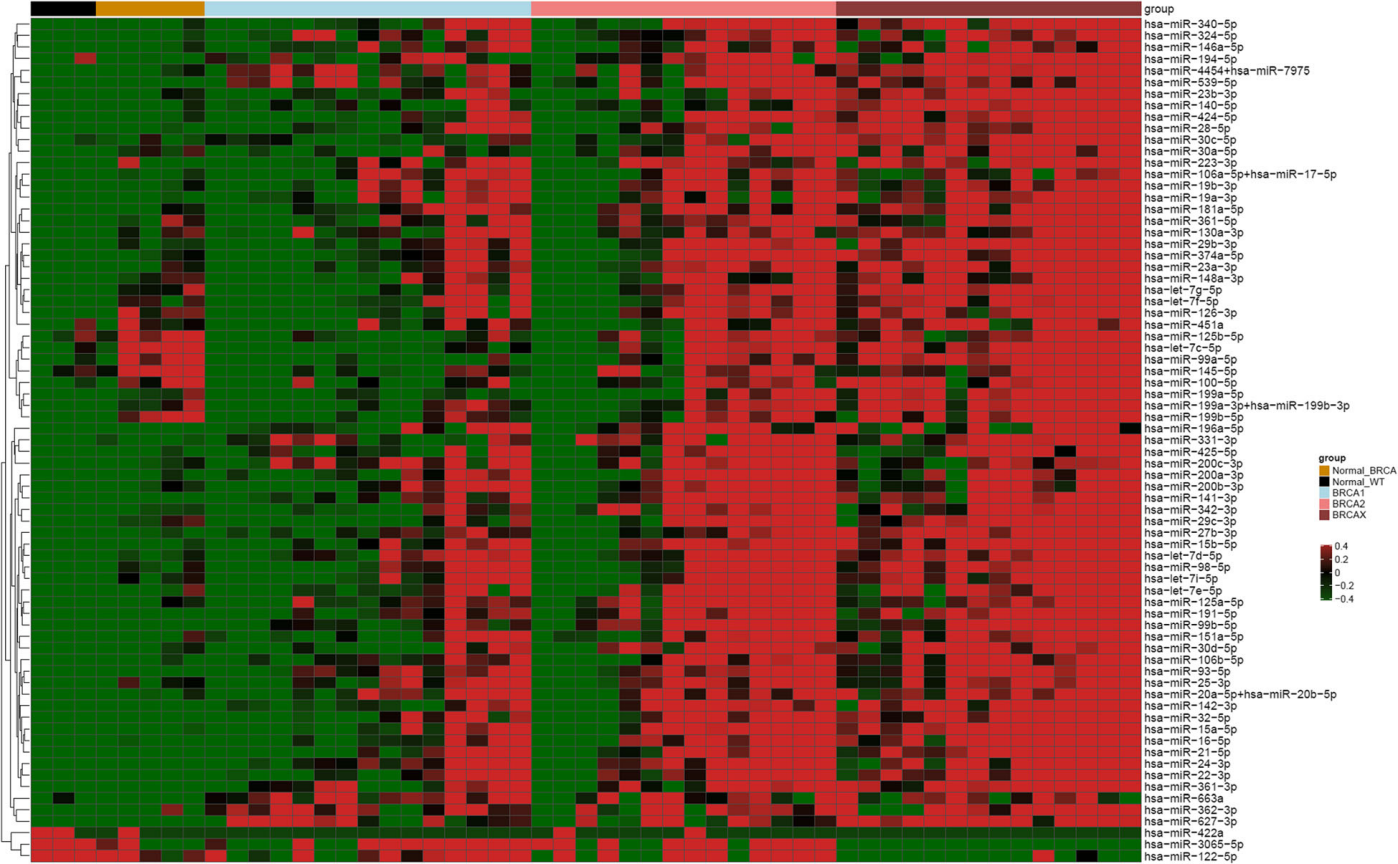
Heat map showing a supervised clustering of differentially expressed miRNAs between NBT and HBC. Each column indicates a sample and each row, a miRNA. Red color indicates upregulation and green, downregulation

In order to explore whether miRNA expression profiling could also discriminate BRCA1, BRCA2, and BRCAX breast tumors, we performed a multiple comparison to identify a miRNA signature among HBC. We found a total of 73 differentially expressed miRNAs, which comprised 70 upregulated and 3 downregulated miRNAs. After a supervised hierarchical clustering analysis, we confirmed that hereditary breast tumors mainly exhibited an upregulated miRNA expression profile as compared to NBT (Fig. 2). We also observed that most BRCA2 breast tumors had expression patterns similar to BRCAX, especially in the upregulated miRNAs cluster, whereas most BRCAX breast tumors exhibited a specific expression pattern in the downregulated miRNAs cluster. Interestingly, we found that some *BRCA1/2*-mutated NBT samples did not present homogenous expression among the NBT groups for specific miRNAs.

Finally, we verified that 8 miRNAs significantly expressed in HBC were commonly deregulated in SBC as compared to NBT (hsa-miR-627-3p, hsa-miR-99b-5p, hsa-miR-539-5p, hsa-miR-24-3p, hsa-miR-331-3p, hsa-miR-663a, hsa-miR-362-3p and hsa-miR-145-5p). Therefore, those miRNAs were excluded from the subsequent analysis.

### miRNAs as biomarkers for hereditary breast tumors

Next, we aimed to identify miRNAs that could be potential diagnostic biomarkers. We identified 25 upregulated miRNAs as potential biomarkers using receiver operating characteristic (ROC) curve analysis. Our results showed higher accuracy in distinguishing HBC from NBT (area under the curve [AUC] > 0.75) in all groups (Table 2). We also observed that these miRNAs achieved slightly higher AUC values in BRCAX breast tumors compared to BRCA1 and BRCA2 breast tumors, suggesting that these miRNAs have higher specificity and specificity rates for hereditary BRCAX as compared to *BRCA1/2*-mutated breast tumors.

**Table 2:** ROC curve analysis for miRNAs as potential biomarkers in hereditary breast cancer

	Normal vs. BRCA1				Normal vs. BRCA2				Normal vs. BRCAX			
microRNA	Sen	Spe	AUC	Cutoff	Sen	Spe	AUC	Cutoff	Sen	Spe	AUC	Cutoff
hsa-miR-28-5p	87%	88%	**0.91**	4.07	78%	88%	**0.84**	4.49	100%	100%	**1.00**	5.37
hsa-miR-361-3p	93%	75%	**0.88**	2.49	71%	100%	**0.89**	3.35	100%	100%	**1.00**	3.38
hsa-miR-93-5p	87%	100%	**0.93**	4.94	86%	100%	**0.90**	4.97	100%	100%	**1.00**	5.91
hsa-miR-32-5p	93%	75%	**0.88**	2.64	93%	100%	**0.93**	3.14	100%	100%	**1.00**	4.24
hsa-miR-191-5p	80%	88%	**0.85**	5.61	86%	100%	**0.94**	6.20	93%	100%	**0.99**	6.11
hsa-miR-27b-3p	87%	75%	**0.88**	3.48	71%	88%	**0.82**	4.02	100%	100%	**1.00**	4.74
hsa-miR-21-5p	60%	100%	**0.81**	7.20	86%	100%	**0.89**	7.11	100%	100%	**1.00**	7.88
hsa-miR-16-5p	73%	88%	**0.87**	6.07	78%	88%	**0.85**	5.92	100%	100%	**1.00**	6.54
hsa-miR-340-5p	67%	88%	**0.83**	3.55	78%	88%	**0.87**	3.55	93%	100%	**0.99**	4.16
hsa-miR-194-5p	100%	63%	**0.81**	1.71	78%	88%	**0.86**	2.78	93%	88%	**0.95**	2.69
hsa-miR-142-3p	80%	100%	**0.93**	6.10	71%	100%	**0.82**	7.39	93%	100%	**0.98**	6.92
hsa-miR-22-3p	80%	88%	**0.91**	3.50	78%	100%	**0.84**	4.13	100%	100%	**1.00**	4.10
hsa-miR-15b-5p	87%	75%	**0.88**	4.50	71%	100%	**0.84**	6.47	100%	100%	**1.00**	6.05
hsa-miR-141-3p	80%	100%	**0.92**	5.36	78%	100%	**0.89**	5.99	93%	100%	**0.93**	5.77
hsa-miR-106b-5p	87%	88%	**0.92**	3.80	93%	75%	**0.88**	3.21	100%	100%	**1.00**	4.49
hsa-miR-425-5p	93%	75%	**0.85**	1.66	86%	88%	**0.95**	2.21	93%	88%	**0.96**	2.43
hsa-miR-4454 + hsa-miR-7975	73%	100%	**0.88**	13.72	71%	88%	**0.82**	13.33	100%	100%	**1.00**	13.88
hsa-miR-196a-5p	73%	100%	**0.87**	4.24	86%	100%	**0.96**	4.29	100%	88%	**0.99**	4.07
hsa-miR-324-5p	80%	88%	**0.88**	3.07	86%	88%	**0.84**	3.29	86%	100%	**0.95**	3.62
hsa-miR-20a-5p + hsa-miR-20b-5p	67%	88%	**0.80**	5.06	71%	100%	**0.83**	5.79	100%	88%	**0.98**	5.01
hsa-let-7d-5p	73%	75%	**0.83**	5.90	71%	88%	**0.81**	6.36	100%	100%	**1.00**	6.64
hsa-miR-19a-3p	67%	100%	**0.81**	3.21	71%	88%	**0.80**	3.02	93%	100%	**0.99**	3.43
hsa-miR-146a-5p	100%	75%	**0.89**	3.78	71%	88%	**0.85**	4.42	86%	88%	**0.95**	4.43
hsa-miR-200c-3p	87%	75%	**0.88**	7.11	71%	100%	**0.87**	7.95	78%	100%	**0.86**	7.78
hsa-miR-106a-5p + hsa-miR-17-5p	60%	100%	**0.81**	4.79	64%	100%	**0.80**	4.84	78%	88%	**0.92**	4.47

*Sen*, sensitivity; *spe*, specificity; *AUC*, area under the curve.

**Fig. 3:**
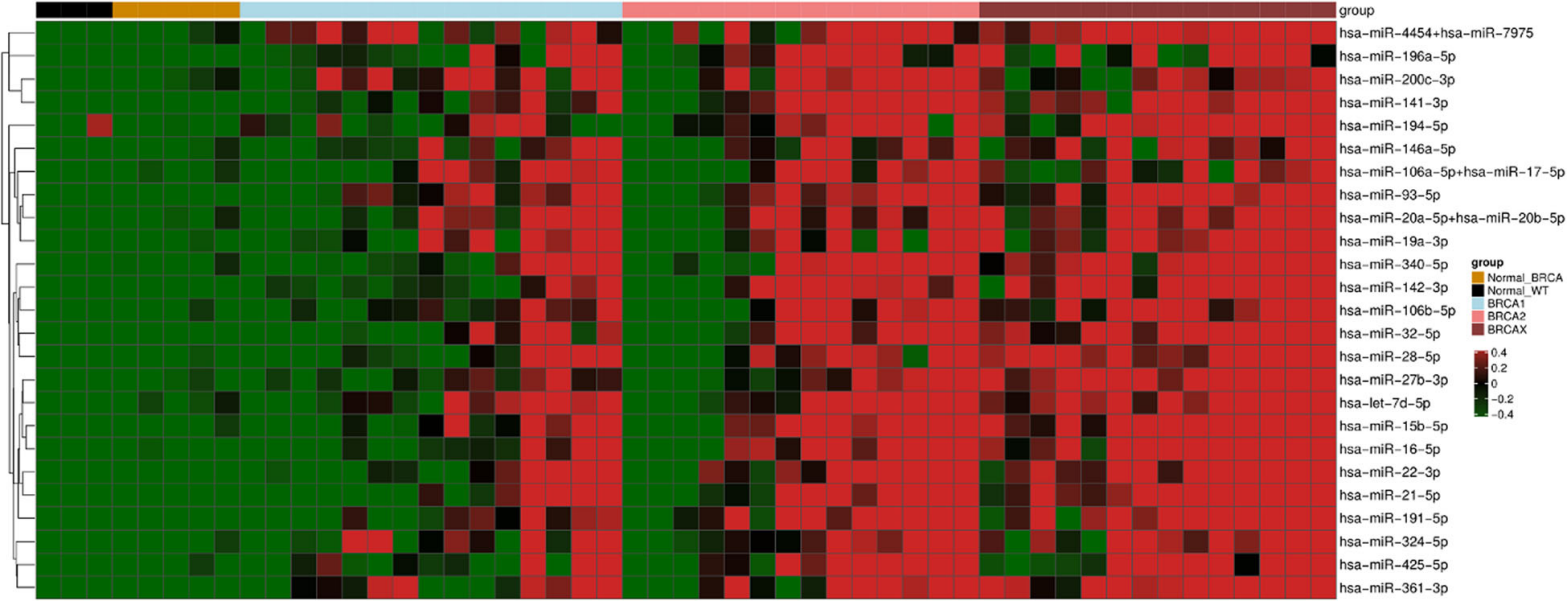
Expression patterns of the best biomarkers according to ROC curve analysis between NBT and HBC. **a** Heat map showing supervised clustering of the best biomarkers. Each column indicates a sample and each row, a miRNA. Red color indicates upregulation and green, downregulation. **b** Expressive upregulated cluster of miRNAs in hereditary breast cancer (especially BRCAX) vs normal breast tissues

Subsequently, we generated a heatmap illustrating the expression patterns of these potential biomarkers across the samples based on a supervised hierarchical clustering analysis (Fig. 3a). Even though most BRCA2 breast tumors presented similar miRNA expression profiles to BRCAX tumors as previously mentioned, we observed that these potential biomarkers had significantly higher mean fold change values among BRCAX samples as compared to BRCA1 and BRCA2 breast tumors (Fig. 3b). Therefore, our findings suggest that these miRNAs could be suitable in discriminating hereditary BRCAX breast tumors from *BRCA1/2*-mutated breast tumors.

### Functional in silico analysis

We further investigated the association of these 25 miRNAs with biological pathways related to carcinogenesis. Previously described genes in breast cancer, such as *TP53*, *PTEN*, and *FOXO1*, were identified as target genes in five main pathways, including ErbB and FoxO signaling, the PI3K-Akt signaling pathway, and miRNAs in cancer and breast cancer. The most significant pathways of the targets associated with breast neoplasm and those with putative roles as oncogenes and TSGs are shown in Table 3.

**Table 3:** Top five pathways related to the best target candidates of miRNAs differentially expressed between normal tissues and *BRCA1/2*-germline mutation carriers and BRCAX cases

Pathway	Genes (targets)	FDR-corrected*P* value
ErbB signaling pathway	AKT2, AKT3, PRKCB, PLCG1, STAT5B, JUN, CDKN1A, CDKN1B, EGFR, NRAS, MAP 2 K4, MAP 2 K1, ABL1, PIK3CB, NRG1, PIK3CA, SRC, CBL, ERBB3, ERBB4, MAPK1, KRAS	2,89E-15
FoxO signaling pathway	AKT2, AKT3, CREBBP, ATM, CDKN1A, CDKN1B, IKBKB, SGK1, EGFR, NRAS, STAT3, STK11, CCND2, CCND1, EP300, MAP 2 K1, FOXO3, FOXO1, SMAD2, SMAD4, SMAD3, BCL6, MDM2, PTEN, PIK3CB, TGFBR2, PIK3CA, MAPK1, KRAS	2,89E-15
MicroRNAs in cancer	TP63, PRKCB, TP53, PLCG1, CREBBP, ATM, EZH2, CDKN1A, CDKN1B, BRCA1, IKBKB, RHOA, EGFR, NRAS, CDKN2A, STAT3, TNC, CCND2, CCND1, PIM1, EP300, MAP 2 K1, FOXP1, CCNE1, NOTCH2, NOTCH1, SOCS1, ABL1, HMGA2, CDK6, MDM2, BCL2, MDM4, FGFR3, PTEN, CASP3, PIK3CA, MET, ERBB3, MAPK1, APC, KRAS	2,89E-15
PI3K-Akt signaling pathway	PPP2R1A, AKT2, MYB, AKT3, KDR, TP53, CDKN1A, CDKN1B, BRCA1, IKBKB, RAC1, JAK1, KIT, SGK1, EGFR, NRAS, TNC, STK11, CCND3, CCND2, CCND1, MAP 2 K1, TSC1, CCNE1, CSF1R, FOXO3, CDK6, MDM2, BCL2, FGFR3, FGFR2, FGFR1, PTEN, PIK3CB, ITGAV, PIK3CA, MET, MAPK1, KRAS	2,89E-15
Breast cancer	RB1, AKT2, AKT3, TP53, JUN, CDKN1A, BRCA1, NCOA1, KIT, EGFR, NRAS, CTNNB1, CCND1, MAP 2 K1, NOTCH2, NOTCH1, ESR1, CDK6, FGFR1, PTEN, PIK3CB, AXIN2, PIK3CA, MAPK1, TCF7L2, APC, KRAS	2,89E-15

*FDR* False discovery rate

## Discussion

In the present study, miRNA expression profiles were analyzed in a series of hereditary breast tumors (*BRCA1/2* and BRCAX-associated breast tumors), sporadic breast tumors and NBT from *BRCA1/2*-germline mutation carriers and non-carriers using NanoString nCounter Technology. Initially, we identified differentially expressed miRNAs that could determine a specific signature of SBC vs. NBT that are related to miRNAs identified in previous studies about sporadic breast tumors (i.e., hsa-miR-145-5p, hsa-miR-429, hsa-miR-137, and hsa-miR-551a) [43–46]. Furthermore, this analysis was important to identify a specific miRNA signature for SBC and to investigate if any miRNAs are shared between SBC and HBC. Thus, we found eight miRNAs (hsa-miR-627-3p, hsa-miR-99b-5p, hsa-miR-539-5p, hsa-miR-24-3p, hsa-miR-331-3p, hsa-miR-663a, hsa-miR-362-3p, and hsa-miR-145-5p) that were also differentially expressed in HBC. These miRNAs were used as a filter to our next analysis with hereditary breast tumors and excluded to allow that we would have a specific miRNA expression profiles of HBC.

We found several differentially expressed miRNAs in HBC compared to NBT with an expressive signature for BRCAX breast tumors. Some of these have been previously described as deregulated in *BRCA1/2*-germline mutation carriers, such as hsa-miR-141-3p; hsa-miR-20a-5p; hsa-miR-21-5p; and hsa-miR-106b-5p [33, 47]. Those miRNAs have also been reported to be deregulated in sporadic breast tumors [33], supporting the hypothesis that some miRNAs could have a relevant role in for both sporadic and hereditary breast cancer carcinogenesis. Furthermore, some of the differentially expressed miRNAs were also found to be deregulated in some *BRCA1/2*-mutated NBT cases, suggesting that NBTs from healthy *BRCA1/2*-germline mutations carriers might display biological alterations due to genomic instability caused by impaired BRCA1 and BRCA2 functions.

We also proposed to investigate whether those differentially expressed miRNAs could be considered as potential biomarkers for discriminate patients harboring *BRCA1/2*-germline mutations from non-carriers. Indeed, many studies investigated the role of miRNAs as diagnostic biomarkers in SBC, but little has been reported in hereditary breast tumors. Although limited in terms of the number of specimens used for miRNA expression profiling, Murria-Estal et al. identified 15 differentially expressed miRNAs that could classify BRCA1, BRCA2, BRCAX and sporadic breast tumors with 75% accuracy. However, miRNAs validated by quantitative polymerase chain reaction (qPCR) (miR-4417 and miR-423-3p) could only discriminate hereditary (BRCA1, BRCA2, and BRCAX) from non-hereditary breast tumors (70.1% accuracy) [31]. On the other hand, Tanic et al. established a biomarker classifier based on six miRNAs that could distinguish *BRCA1/2*-mutated from non-mutated breast tumors with 92% accuracy [32]. Both studies were primarily based on microarray technology for the screening of differentially expressed miRNAs, a laborious technique that requires complementary DNA (cDNA) synthetized from highly stable messenger RNAs (mRNA) – which are rarely obtained from FFPE tissues and other low-quality samples – and experimental validation by qPCR. In the present study, we assessed miRNA expression profiles in FFPE samples using NanoString technology – a high throughput, rapid, reproducible and sensitive platform for molecular quantification that does not require target sequence amplification and technical replicates [48–51]. All samples have accurate results using NanoString technology. We found 25 upregulated miRNAs that could classify HBC (especially BRCAX breast tumors) with high accuracy rates according to ROC curve analysis (AUC: ≥0.80). Because BRCAX patients have been unnecessarily referred to *BRCA1/2*-germline mutation testing [19–22], we assume that these miRNAs could identify those patients that might not benefit from genetic testing and personalized therapies, such as platinum-based chemotherapy and PARP inhibitors [34].

Finally, in silico pathway analysis identified several common target genes involved in breast cancer carcinogenesis of the 25 miRNAs identified as potential biomarkers for *BRCA1/2*-germline mutation carriers and BRCAX patients using ReactomeFIViz. These genes are associated with important pathways, including ErbB and FoxO signaling, the PI3K-Akt signaling pathway, and miRNAs in cancer and breast cancer. However, because BRCAX breast tumors were also included in the HBC group, we believe it is a limitation of the present study, and further studies are needed to investigate target genes and signaling pathways specifically deregulated in *BRCA1/2*-mutated breast tumors. The findings of this work showed that miRNA signatures could serve as potential biomarkers to discriminate HBC that could improve the low specificity rates of the models for *BRCA1/2*-mutation prediction [19–22]. Further studies are necessary to evaluate the inclusion of new miRNAs biomarkers as additional parameters in the available prediction models to provide a better selection of patients that should proceed to BRCA1/2 genetic testing.

## Conclusions

In conclusion, this work provides the first evidence of a molecular profile of miRNAs that could discriminate with high accuracy *BRCA1/2*-germline mutation carriers and BRCAX from NBT in Brazilian women using NanoString technology. Furthermore, these miRNAs could have potential value as a complementary clinical diagnostic tool to identify breast cancer patients that could benefit from *BRCA1/2*-mutations genetic testing and personalized clinical management. However, further larger prospective studies are required to validate these profiles.

## Data Availability

All data used and analyzed during this study are available from the corresponding author on reasonable request.

## Abbreviations

AUC: Area under the curve
ER: Estrogen receptor
FDR: False discovery rate
FFPE: Formalin-fixed paraffin-embedded
HBC: Hereditary breast cancer
HBOC: Hereditary breast and ovarian cancer syndrome
HER2: Human epidermal growth factor receptor 2
miRNA: MicroRNA
NBT: Normal breast tissue
PR: Progesterone receptor
qPCR: Quantitative polymerase chain reaction
ROC: Receiver operating characteristic
SBC: Sporadic breast cancer
TSG: Tumor suppressor gene
WT: Wild type
